# Supplementary figures and images for: Characterisation of the spectrum and genetic dependence of collateral mutations induced by translesion DNA synthesis
Source: PLoS Genet. 2022 Feb 7;18(2):e1010051. doi: 10.1371/journal.pgen.1010051 (PMC8870599; doi:10.1371/journal.pgen.1010051)

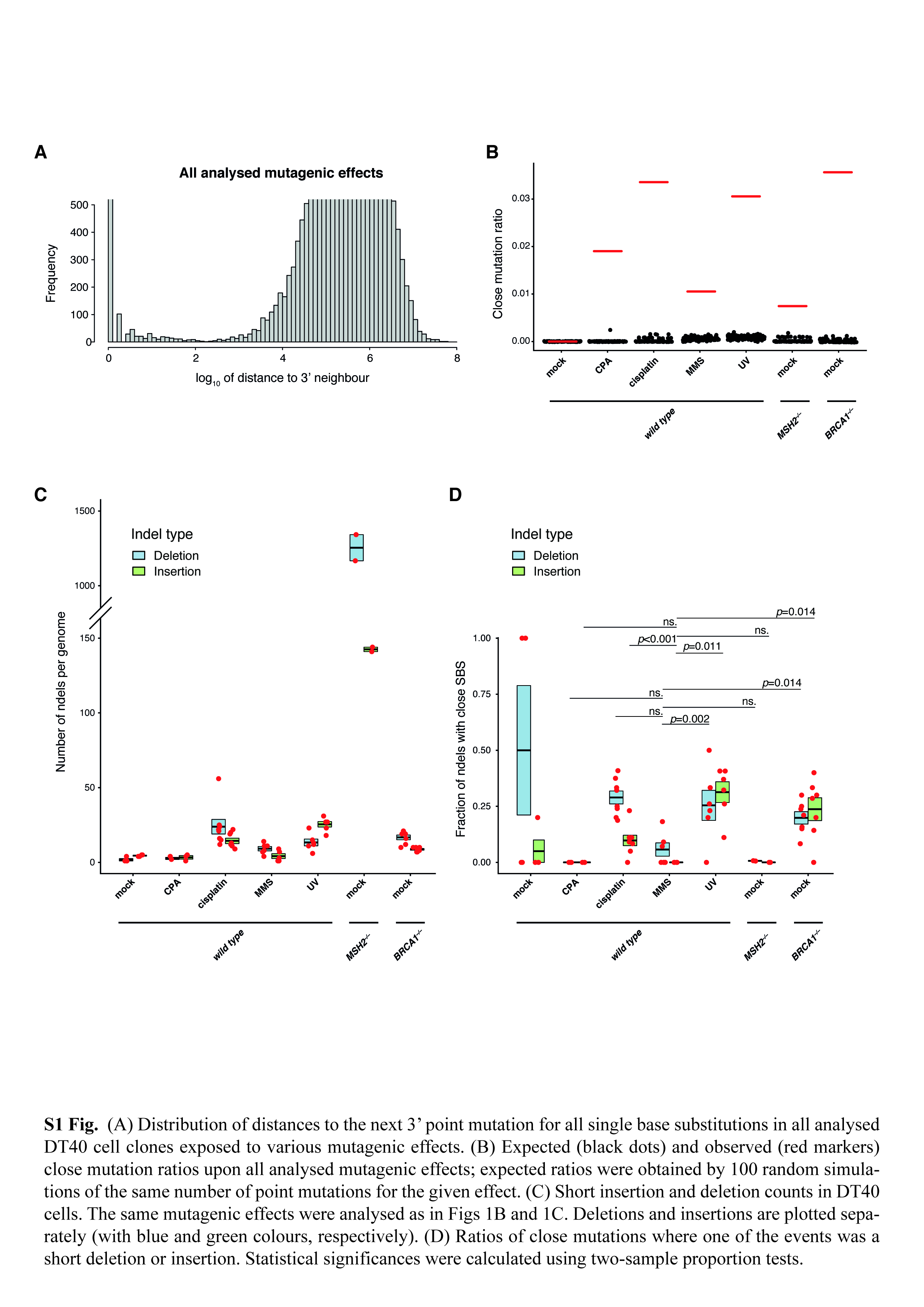

Supplement: S1 Fig — (A) Distribution of distances to the next 3’ point mutation for all single base substitutions in all analysed DT40 cell clones exposed to various mutagenic effects. (B) Expected (black dots) and observed (red markers) close mutation ratios upon all analysed mutagenic effects; expected ratios were obtained by 100 random simulations of the same number of point mutations for the given effect. (C) Short insertion and deletion counts in DT40 cells. The same mutagenic effects were analysed as in Fig 1B and 1C. Deletions and insertions are plotted separately (with blue and green colours, respectively). (D) Ratios of close mutations where one of the events was a short deletion or insertion. Statistical significances were calculated using two-sample proportion tests. (TIF) [file pgen.1010051.s001.tif]

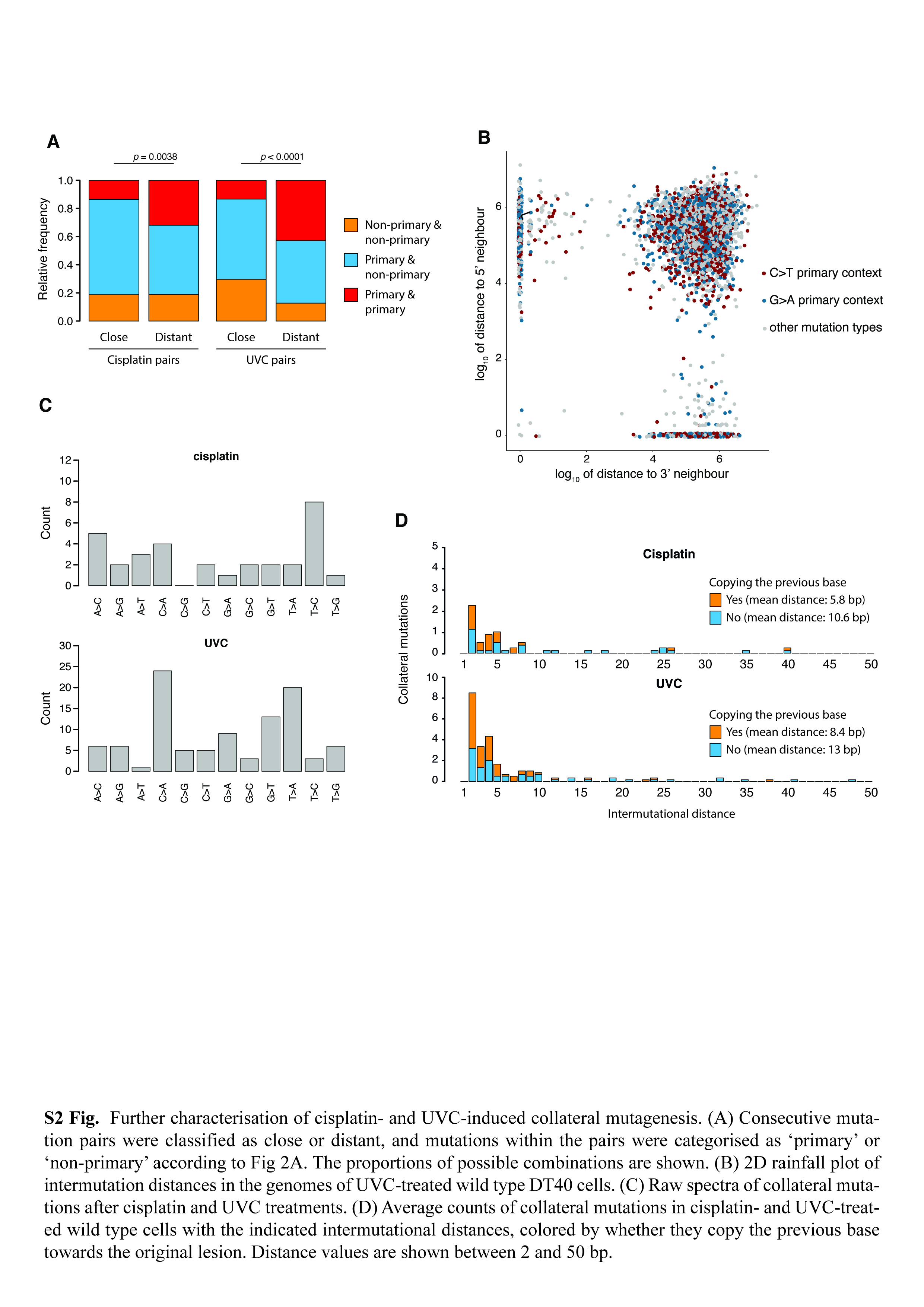

Supplement: S2 Fig — (A) Consecutive mutation pairs were classified as close or distant, and mutations within the pairs were categorised as ‘primary’ or ‘non-primary’ according to Fig 2A. The proportions of possible combinations are shown. (B) 2D rainfall plot of intermutation distances in the genomes of UVC-treated wild type DT40 cells. (C) Raw spectra of collateral mutations after cisplatin and UVC treatments. (D) Average counts of collateral mutations in cisplatin- and UVC treated wild type cells with the indicated intermutational distances, coloured by whether they copy the previous base towards the original lesion. Distance values are shown between 2 and 50 bp. (TIF) [file pgen.1010051.s002.tif]

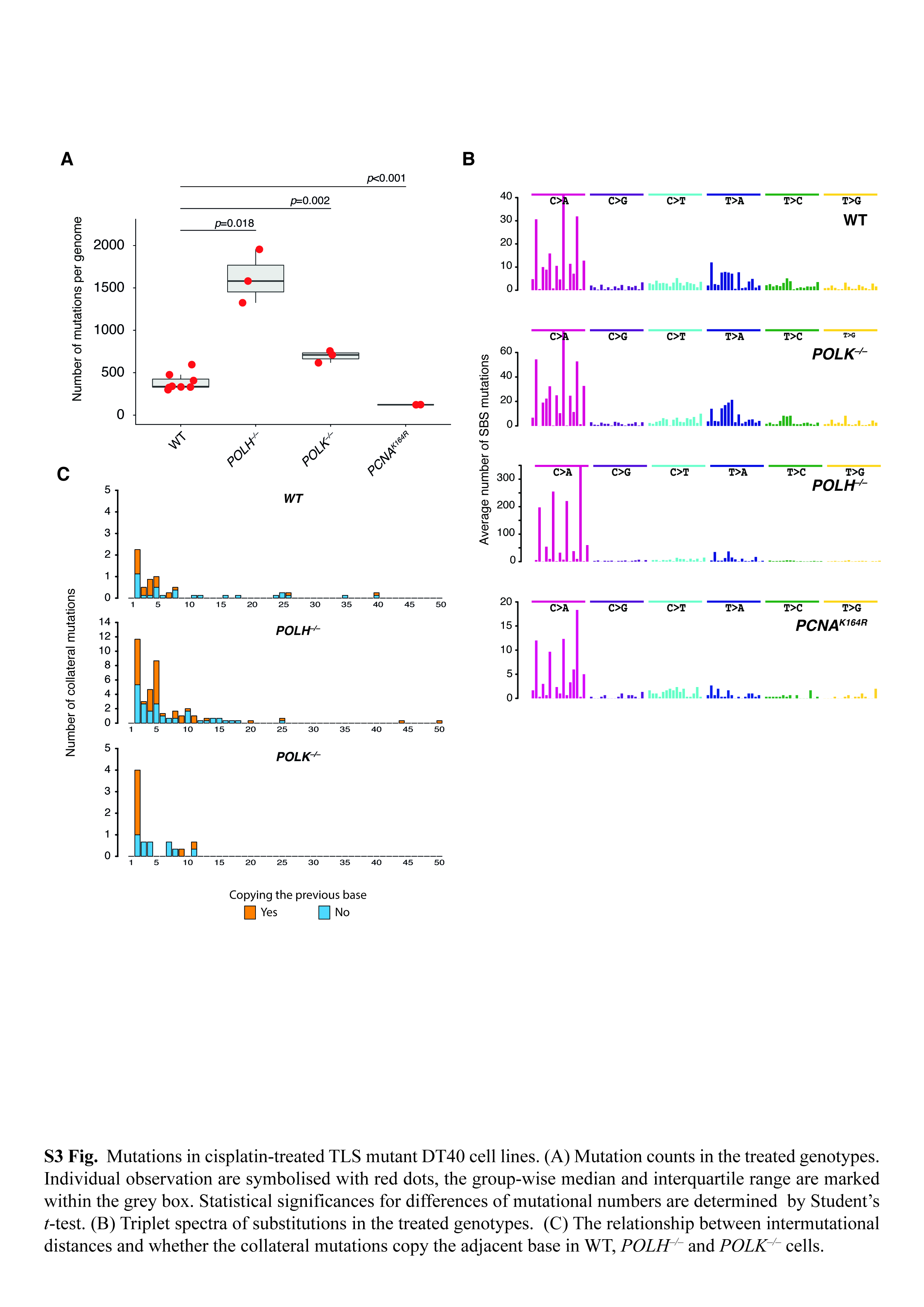

Supplement: S3 Fig — (A) Mutation counts in the treated genotypes. Individual observations are symbolised with red dots, the group-wise median and interquartile range are marked within the grey box. Statistical significances for differences of mutational numbers are determined by Student’s t-test. (B) Triplet spectra of substitutions in the treated genotypes. (C) The relationship between intermutational distances and whether the collateral mutations copy the adjacent base in WT, POLH–/– and POLK–/– cells. (TIF) [file pgen.1010051.s003.tif]

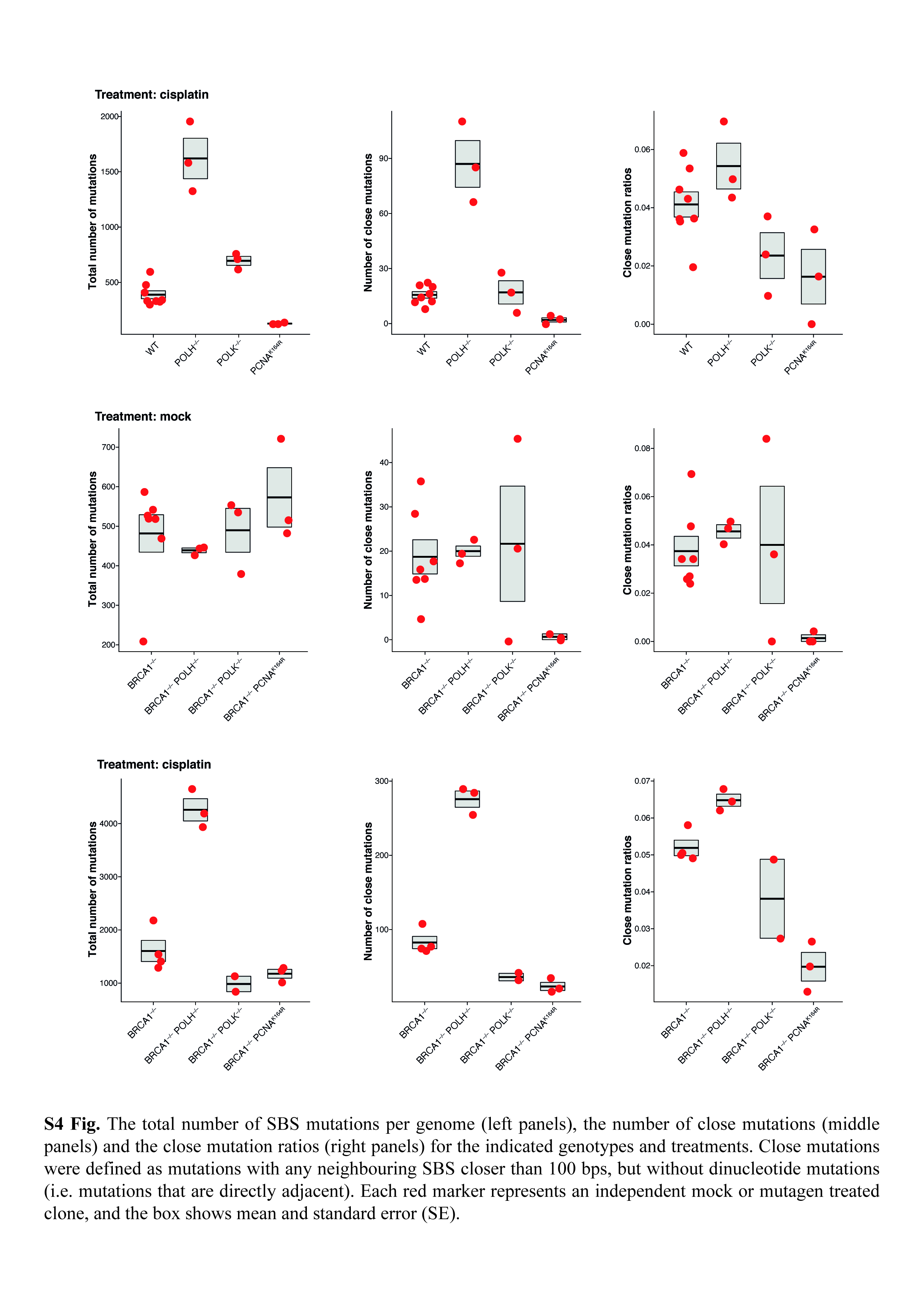

Supplement: S4 Fig — The total number of SBS mutations per genome (left panels), the number of close mutations (middle panels) and the close mutation ratios (right panels) for the indicated genotypes and treatments. Close mutations were defined as mutations with any neighbouring SBS closer than 100 bps, but without dinucleotide mutations (i.e. mutations that are directly adjacent). Each red marker represents an independent mock or mutagen treated clone, and the box shows mean and standard error (SE). (TIF) [file pgen.1010051.s004.tif]

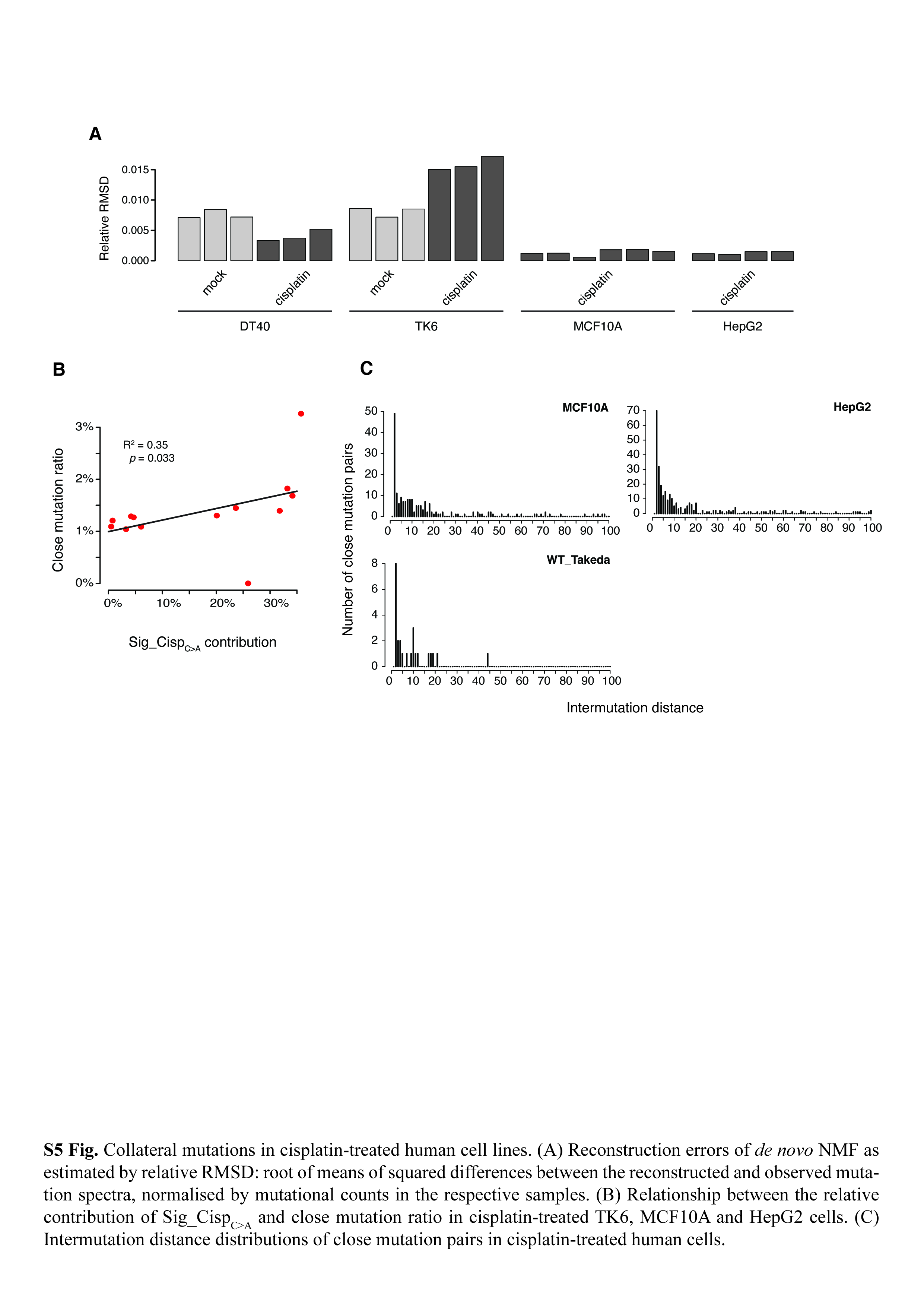

Supplement: S5 Fig — (A) Reconstruction errors of de novo NMF as estimated by relative RMSD: root of means of squared differences between the reconstructed and observed mutation spectra, normalised by mutational counts in the respective samples. (B) Relationship between the relative contribution of Sig_CispC>A and close mutation ratio in cisplatin treated TK6, MCF10A and HepG2 cells. (C) Intermutation distance distributions of close mutation pairs in cisplatin-treated human cells. (TIF) [file pgen.1010051.s005.tif]

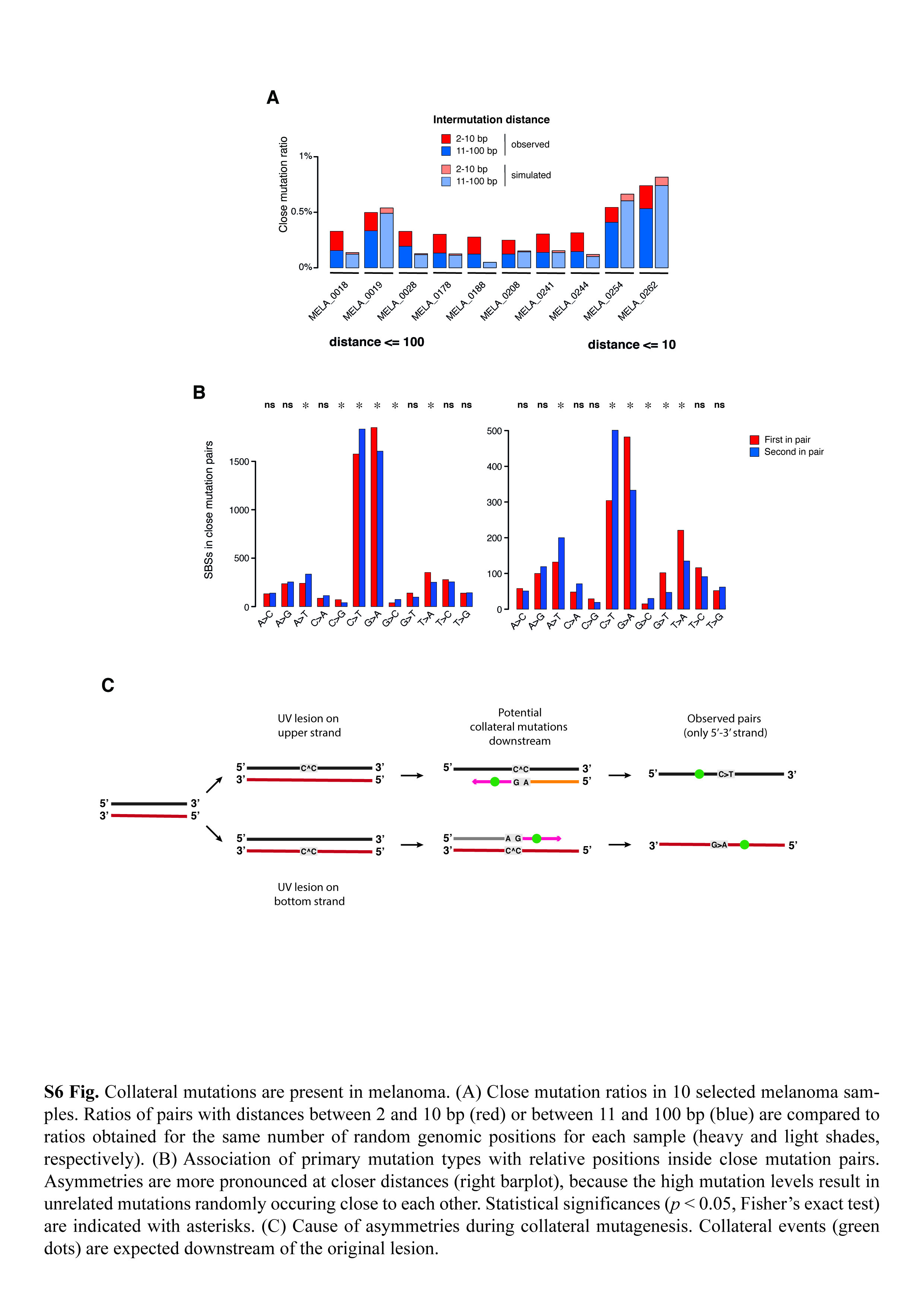

Supplement: S6 Fig — (A) Close mutation ratios in 10 selected melanoma samples. Ratios of pairs with distances between 2 and 10 bp (red) or between 11 and 100 bp (blue) are compared to ratios obtained for the same number of random genomic positions for each sample (heavy and light shades, respectively). (B) Association of primary mutation types with relative positions inside close mutation pairs. Asymmetries are more pronounced at closer distances (right barplot) because the high mutation levels result in unrelated mutations randomly occurring close to each other. Statistical significances (p < 0.05, Fisher’s exact test) are indicated with asterisks. (C) Cause of asymmetries during collateral mutagenesis. Collateral events (green dots) are expected downstream of the original lesion. (TIF) [file pgen.1010051.s006.tif]

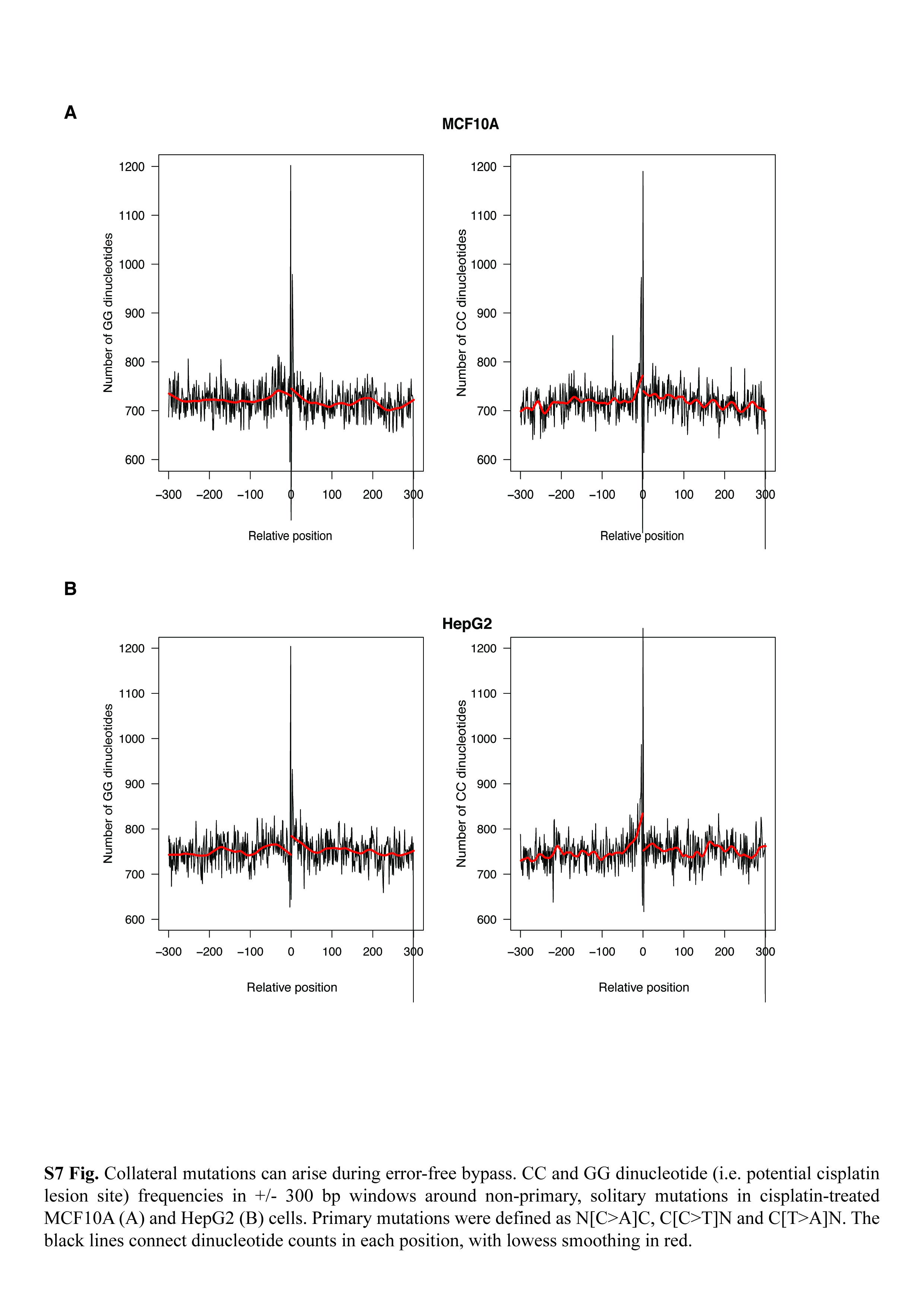

Supplement: S7 Fig — CC and GG dinucleotide (i.e., potential cisplatin lesion site) frequencies in +/- 300 bp windows around non-primary, solitary mutations in cisplatin-treated MCF10A (A) and HepG2 (B) cells. Primary mutations were defined as N[C>A]C, C[C>T]N and C[T>A]N. The black lines connect dinucleotide counts in each position, with lowess smoothing in red. (TIF) [file pgen.1010051.s007.tif]

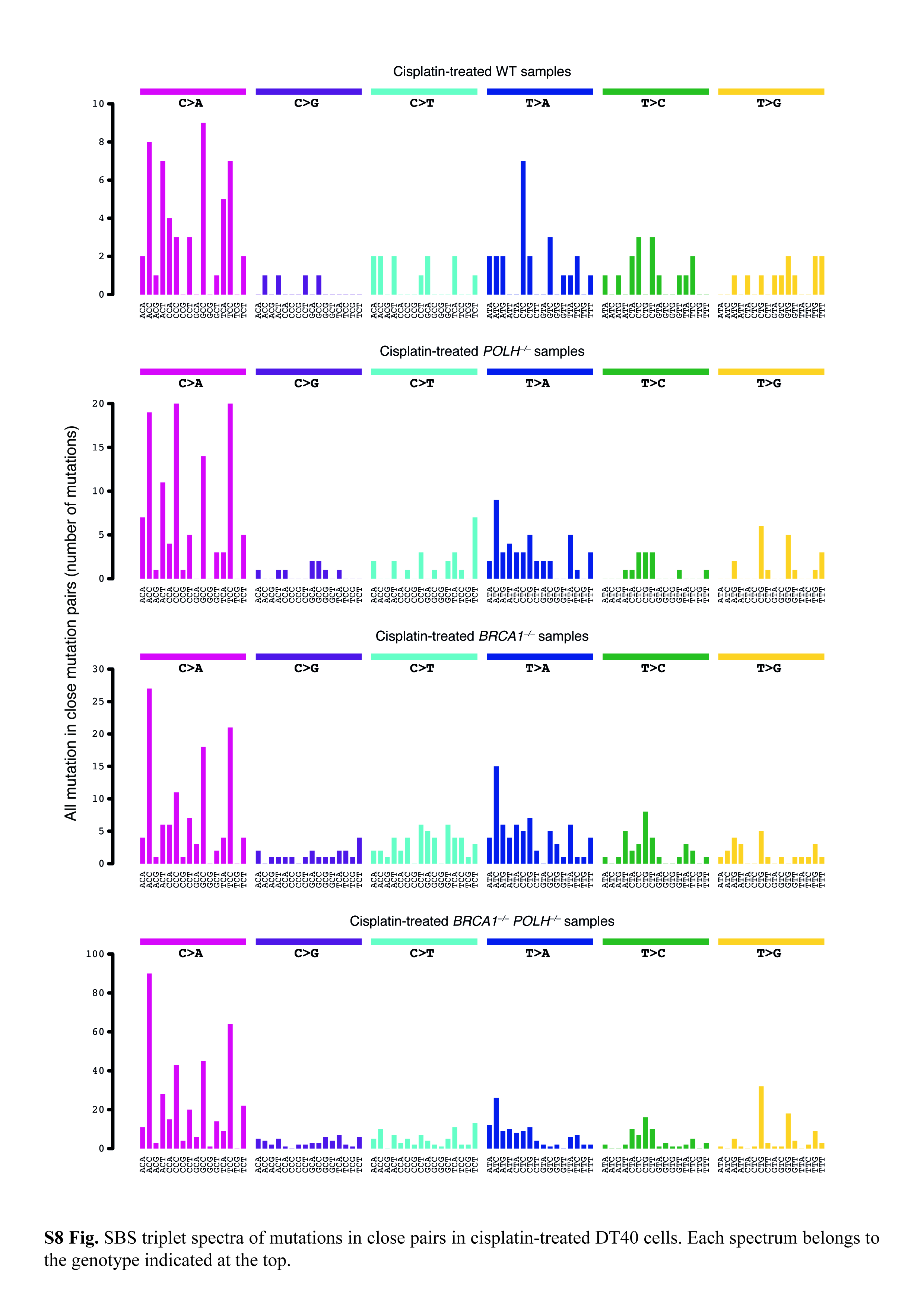

Supplement: S8 Fig — Each spectrum belongs to the genotype indicated at the top. (TIF) [file pgen.1010051.s008.tif]
