# Supplementary material for: Characterisation of the spectrum and genetic dependence of collateral mutations induced by translesion DNA synthesis
Source: PLoS Genet. 2022 Feb 7;18(2):e1010051. doi: 10.1371/journal.pgen.1010051 (PMC8870599; doi:10.1371/journal.pgen.1010051)
Supplement: Attachment — Submitted filename: Response to reviewers.docx [file pgen.1010051.s011.docx]

**Response to reviewers**

We would like to sincerely thank all reviewers for critically reading our manuscript and making numerous suggestions for improvement. We believe that we have managed to address all their concerns, and in the process significantly improve the manuscript. Our point-by-point responses are shown below.

**Reviewer #1:** Póti et al characterized a process they refer to as collateral mutagenesis. They find that in mammalian cells, some mutagenesis processes (related to cisplatin, UVC radiation, defects in homologous recombination pathways) create not only a primary mutation but a second one close by, within 100 base pairs of the first. The primary mutation is easy to identify for these two mutagens but may be more difficult, for example for deficiencies in BRCA1. They characterized this process in several cell systems and primary tumors and identify some of the mechanisms of collateral mutagenesis such as an at least partial requirement for ubiquitinylated PCNA and DNA polymerase kappa. This is a very interesting paper and warrants publication. However, I have several comments or questions:

1) How much of a concern are sequencing artefacts for interpretation of all data? Another technical question is: Were all the mutation pairs identified on the same DNA sequencing reads?

Closely clustered alterations are indeed one of the major signs of sequencing artefacts, caused either by erroneous readouts during the sequencing reaction, or by the inherent repetitive or ambigous nature of certain genomic regions. Our experimental approach, however, helps us filter out the majority of such events. We sequence single cell clones and only aim to detect highly confident clonal heterozygous alterations, supported by usually more than 5 read pairs, which eliminates sequencing errors. The variant calling tool used in this study, IsoMut, is also highly effective in filtering false positives arising due to alignment ambiguity, as these are expected to be systematic errors arising in many samples, and this software will only output unique to one sample upon analysing around 30 samples concurrently. IsoMut was designed to provide a tunable false positive rate, thus we can guarantee near zero false positives by setting the filtering parameters such that ancestral clones have no more than 5 unique SBS mutations or one indel. We have included this information in the Methods, and also provided more information on the post-filtering step aimed at removing false positives due to interspecies sequence contamination that often occurs at sequencing centres.

We thank the reviewer for the question regarding the phasing of close mutation pairs. We have now verified that all pairs in DT40 cells were found on the same sequencing reads, which is in agreement with the idea of collateral mutagenesis. This information is now included in the second paragraph of the Results and in the Methods.

2) What do the authors think, why MMS doesn’t produce collateral mutations? Could it be that these collateral mutations are induced by NER gap filling rather than by a DNA damage bypass polymerase? This possibility also comes to mind because most dual mutations were separated by at most 10 bp for cisplatin and UVC.

In response to question 5, we included a new supplementary figure panel which shows that the number of close mutation pairs is higher than expected by chance in case of all treatments as well as the mock treated control (Fig. S1B) In the text we emphasise that “only cisplatin, UVC treatments and the lack of BRCA1 caused significantly higher close mutation ratios“, but we added a comment that ‘Notably, CMR was above the values expected by chance in all treated or mutant samples”.

The lower ratio of collateral mutations in MMS treated cells can be most probably explained by the differences in the chemical structures of the lesions. MMS-generated lesions are single methyl adducts, *in vitro* mainly on the N7 of guanine, but to a lesser extent also on N1 and N3 of adenine and on the DNA backbone (Beranek, Mutat Res, 1990). Similar lesions were also detected in MMS treated yeast cells (Mao et al, Genome Res, 2017). Even though in these studies 8-10 times more guanines were found to be affected than adenines, still the majority of mutations in MMS treated DT40 cells occurred at A/T bases, suggesting that methyladenines are more mutagenic. It is possible that the polymerase responsible for synthesis over these lesions is less prone to collateral mutagenesis - perhaps the replicative polymerases are partly responsible. The inactivation of PCNA ubiquitylation did not decrease MMS-induced mutagenesis in our previous, cited study (35, Zamborszky et al. 2017), suggesting a limited role for TLS. We did not insert this speculative discussion into the text.

We believe that the profound effect that TLS KOs had on close mutation ratios clearly demonstrates that the TLS rather than NER pathway is responsible for these events. Of course, as we noted in the Discussion, it doesn’t exclude the possibility that TLS is performed in post-replicative gaps, where the length of the gaps can limit the possible distance of the TLS tract.

3) The authors discuss UVC-induced mutations and their similarity to the COSMIC mutation signature 7b in terms of C>T and T>A mutations. However, SBS7b (or SBS7a) have almost no mutations of the type T>A.

Whereas the UVC-induced mutational spectrum in DT40 cells was quite similar to SBS7a or SBS7b, the COSMIC signatures most often found in sun-exposed cancers, the reviewer correctly points out that they were not identical. The difference is indeed mainly due to the lack of T>A mutations in the signatures, but the relative proportions of the C>T categories also differ, for example, C[C>T]T and T[C>T]T peaks are much less prominent in the COSMIC patterns. This discrepancy, similarly to the differences in close mutation ratios between UV-C treated DT40 cells and melanoma samples may be caused by both cell type specific availabilities of TLS factors and by the different UV subtypes involved (UV-C in our experiments vs. UV-A and UV-B in sunlight). The hypothesis that the arising T>A mutations are of primary nature is supported by their dipyrimidine context and by their orientation bias in close mutation pairs, as shown on Fig. 2C and discussed in the text.

4) For the published data from melanoma samples, how were these samples selected (only 10 were used)?

Our criteria for the selection of melanoma samples were the following:

* cutaneous subtype

* primary tumour

* total mutation counts should cover the whole range of possible values

We have added this information to the main text.

5) The authors present no clear statistical analysis to determine if the close mutation pairs occur at frequencies greater than expected by chance.

To show this, we simulated the same number of mutations randomly on the Galgal4.73 genome as detected for each mutagenic effects shown on Fig1B-C, and calculated the close mutation ratios for each of 100 simulation rounds. As shown in the new FigS1B, these ratios were in every case lower than the observed values, unlike in melanoma samples, where due to sunlight-derived hypermutagenesis mutations also randomly occur close to each other, as shown in FigS5A.

6) That authors mention that they were unable to perform treatments for REV1–/– and REV3L–/– mutants, as 10 μM cisplatin was lethal for these cells. However, they could have used a lower dose of cisplatin.

We share the reviewer’s interest in the contribution of REV1 and REV3 to collateral mutagenesis. We have indeed attempted to perform the equivalent treatments with lower cisplatin concentrations. However, we found that these cells are two orders of magnitude more sensitive than the wild type (cytotoxicity curves for 1 h treatments of WT and REV1-/- are shown below), so instead of 10 μM we could only use 100 nM treatments. If such a treatment elicited 100x fewer lesions and primary mutations, then we would expect only about 3 cisplatin-induced primary mutations and no collateral mutations in each sequenced genome. We therefore had to forgo this experiment.

7) In melanoma genomes, around 0.25% of the mutations belonged to the close mutation pairs. This number seems much smaller than the authors' own UVC mutation data with a cell line where those mutations seemed to be around 3% of all mutations. How do the authors explain such a large difference?

In the Discussion, we mentioned several possible reasons for this discrepancy. First, DT40 cells were treated with UVC, while skin cancers are rather affected by the UVA and UVB components of sunlight. These subtypes differ in the wavelength of the radiation, which means that they represent different energy levels, and it has been shown for example, that solar radiation does not induce (6-4) photoproducts (Yoon et al, J Mol Biol, 2000), but almost exclusively CPDs. CPDs and (6-4)PPs require different polymerases for bypass (Varga et al, PLoS One, 2012; Szüts et al, NAR, 2008), with possibly different tendencies to form collateral mutations: for example, CPDs are often bypassed by polymerase eta, and based on our results with cisplatin, it seems that this enzyme does not generate secondary mutations.

A likely additional explanation, also presented in the discussion, is that crosslinked bases inside a CPD are especially prone to deamination [ref. 55]. It means that a major population of CPDs will contain uracils instead of cytosines, and even if polymerase eta or delta bypasses them “correctly”, they will result in point mutations. Thus the majority of the C>T changes in CPD-loaded melanoma precursors are not caused directly by mutagenic TLS, explaining the low number of collateral events even with a very high number of solitary mutations.

8) Regarding SBS3 in BRCA1-deficient cells, SBS3 has a rather heterogenous, almost random distribution of mutations. Could this signature be an artifact? It may be difficult to assign a specific pattern to SBS3.

The reviewer is absolutely correct that the flat nature of COSMIC SBS3 makes it hard to draw any conclusions about its exact origin or the associated mechanisms. However, this signature is one of the best established COSMIC patterns, as it was already found in the first version of the database, and it is consistently detected as a hallmark of BRCA1 or BRCA2 mutated cancers. Our research group also has already shown that it is causatively linked to the lack of any of a large group of HR factors (ref. 25, Póti et al, 2019). In response to a referee request, in that paper we also demonstrated that SBS3 can be reliably differentiated from the similarly broad-spectrum SBS5 in a mixed spectrum (Fig. S5, Póti el at, 2019)

9) The part on collateral mutagenesis on a presumably correctly bypassed lesion is a little bit more speculative. If the substitutions are not paired, then how do you know that this is collateral mutagenesis?

We prefer to think of the reasoning as deductive rather than speculative. On Fig5B, we showed that in the vicinity of unpaired non-primary point mutations, CC and GG dinucleotides (which are potential cisplatin lesion sites) are more often present than their long-range overall frequency. The asymmetry of their occurrence makes it convincing that these would be cisplatin lesion sites: GG are enriched in the 3’ direction, whereas CC are enriched in the 5’ direction. We have considerably rephrased the presentation of these data in the revised manuscript and included calculations of significance.

10) Figure S5B:

When the authors display the mutation type for the first in pair and second in pair mutations, they look similar. But according to the author's conclusion, the second mutation should be more of a random type. Also, does the first mutation correspond to dipyrimidine positions?

The data is not consistent with Fig. 2C.

In melanoma samples, the majority of close pairs are not proper collateral events, but are due to the sunlight-caused hypermutagenic process in these cancers. This means that in many close pairs both mutations are primary, which reduced the asymmetry arising from the primary-collateral pairs. The overall mutagenic spectrum is strongly dominated by the mutagenic effect of sunlight, therefore the randomly arising primary-primary close pairs reproduce the overall spectrum.

In Fig2C, the expected contribution of primary-primary close pairs was virtually zero (see the new fig. S1D), so the signal of the collateral processes was much clearer. The panel on the right side of FigS5B is more similar to Fig2C (although still noisy), exactly because within shorter distances the true signal from collateral mutations becomes apparent even in the hypermutated melanoma samples.

We have updated the legend of Figure S5 to make this clearer.

11) Figure S6:

I don't understand this Figure. This is showing CC or GG frequencies around non-primary solitary mutations. However, there is a huge spike of GG/CC at the zero-position suggesting a primary event.

The zero value on the X-axis of Fig. S6 refers to the positions of the non-primary, solitary mutations. As a result, the base or dinucleotide frequencies in the center of the plots will be entirely determined by the triplet context of these substitutions. The excluded primary mutation types were N[C>A]C, C[C>T]N and C[T>A]N, and as it can be seen for example on Figure 1A and 1B of the paper originally describing these treated cells (Boot et al, Genome Research, 2018), even after removing the primary events the remaining mutation classes were mostly centered on cytosines, mainly on other C>A and C>T categories, causing the peaks in the middle part of FigS6. In other words, some of the mutations we classified as non-primary may in fact belong to rare classes of primary mutations. Excluding these would likely make the enrichment of nearby GG positions stronger, but we opted for a conservative approach when classifying primary mutations.

Minor points:

Fig. 2F:

"Collateral mutations tend to copy their immediate neighbour"

Please explain this a little better. Does it mean that the process would create dinucleotides of the same type, such as AA? If so, why would this be the case?

By “copying their immediate neighbour”, we meant that the inserted nucleotide will be the one that would have been appropriate for the preceding template base, from the point of view of the polymerase. This phenomenon is also known as “template slippage” (Kunkel and Soni, J Biol Chem, 1988), and it will indeed result in dinucleotides of the same type. We clarified this at the first mention in the Results.

References 24-26 and possibly others:

These references are incomplete.

We apologise for the incomplete references, the citations have been corrected.

**Reviewer #2:** The manuscript “Characterization of the spectrum and genetic dependence of collateral mutations induced by translesion DNA synthesis” evaluates the production of closely-spaced mutations in chicken DT40 and human cells. The authors utilize whole genome sequencing, primarily of DT40 cells, to identify mutations associated with cisplatin treatment, UVC treatment, or homologous recombination (HR) deficiency. They determine that these treatments and HR-deficiency induce characteristic base substitution signatures. Among these mutations, they also find evidence of closely spaced mutations (i.e. multiple mutations within 100 bp of each other). Based upon the characteristic base substitution patterns of cisplatin and UV treatment, the authors determine that these mutation pairs predominantly consist of a primary mutation resulting from mutagenic bypass of a damaged base, and a secondary, downstream mutation that does not fit the characteristic signature of the mutagen and therefore is likely due to TLS synthesis making mistakes on an undamaged template. The authors confirm that these collateral mutations are caused by TLS polymerases as they are less prevalent in DT40 cells with a PCNA K164R mutant. A role for pol Kappa in generating collateral mutations in response to cisplatin is also observed. Similar patterns of collateral mutations are observed in cisplatin-treated human cancer cell lines and in UV exposed melanoma genomes, suggesting that similar mechanisms observed in DT40 cells also operate in human cells. Finally, the authors attempt to find evidence that TLS recruitment to DNA lesions can result in the secondary mutations (i.e. mutations at the damage site) during error-free bypass of a DNA lesion and estimate the contribution of these types of events to the overall mutation load.

In general, the sequencing assays and analyses are well executed and the data within the manuscript appears to support a role of TLS in generating collateral mutations during lesion bypass in chicken and human cells. Similar induction of collateral mutations by TLS in E. coli and yeast is well known. Additionally, a recent publication has shown a reduced fidelity variant of pol zeta can mediate collateral mutations during site specific bypass of a benzo[a]pyrene in a plasmid substrate in human cells. As such, the confirmation that TLS induces collateral mutations in chicken genomes exposed to DNA damaging agents is not surprising. The manuscript does, however, provide a few additional pieces of mechanistic information on which polymerases contribute to collateral mutagenesis in higher eukaryotes. I have the following specific critiques.

1) In Figs 1c, 2d, 4a, 4b, 4d, 4e, S4b, and S5a the number of close mutation pairs appears to be standardized by the total number of mutations in the sample (i.e., close mutation ratios are reported and analyzed statistically). This is potentially problematic as changes in solitary mutation numbers could impact this ratio and the statistical results. Therefore, the number of close mutation pairs per genome should be presented and statistically evaluated to determine if the occurrence of collateral mutations is changing dependent on genotype or treatment.

We presented close mutation ratios to observe their formation relative to the primary mutagenic process.

The majority of the mutations in the analysed genomes result from TLS (except in MSH2-/- cells), so by normalising close mutations to overall mutation numbers, we observe and present collateral mutagenesis relative to TLS events. We think this is a scientifically prudent approach, as collateral mutagenesis is a product of the TLS process. The overall number of mutagenic TLS events and close mutation pairs would be affected by many confounding factors such as the dose of the treatment used, or the toxicity of the treatment. For example, equitoxic cisplatin treatment resulted in 10x fewer mutations than UVC treatment, but the close mutation ratios were very similar. Also, e.g. in Figure 4E the cisplatin-treated samples have many more mutations than the mock treated samples in 4D, but their CMRs are handily comparable. Detailed mutation numbers are provided in the supplementary material if any reader wishes to perform further analysis, but we think it would be confusing to show and perform statistics on overall numbers of close mutation pairs.

2) In Fig. 2F, the authors state that collateral mutagenesis during cisplatin-lesion bypass involves a slippage mechanism and copying the upstream base. This appears to be true for cisplatin-induced mutations, however, no statistics are performed. Moreover, the authors indicate that this is a general mechanism as a similar pattern is seen in UVC treated cells. However, for UVC treated cells, this appears to primarily be true for N>A mutations and is not true for N>C and N>T mutations. Without having a statistical evaluation of the similarity between the cisplatin and UV treated cells, this is an overstatement.

The patterns between the cisplatin- and UVC-induced collateral mutations on Fig2F are indeed different, mainly because the local sequence context in the vicinity of cisplatin- and UVC-lesions is also different (see the rightmost group of bars on the modified Fig2F). We obtained this latter pattern by selecting a random set of solitary primary mutations a hundred times (with a set size equal to the number of analysed close pairs), and collecting the sequences in in their vicinities, using the distance distribution observed for mutation pairs. Even though the distribution of the adjacent bases was not identical to the simulated background, we can see the bias towards C and T bases for the regions around cisplatin, and towards A and G bases for UVC. The difference may be attributed to the specificities of the actual polymerases.

Although the patterns themselves are quite distinct, the underlying rule is the same: the variant allele for each event more often matches the previous base towards the original lesion. We showed that this association is significantly different from what could be expected by using Fisher’s exact test to compare the observed counts of events showing or not showing slippage vs the expected ratio, which is 1/3 and 2/3 (because the variant allele of a point mutation can be any of three bases). These comparisons were significant for both cisplatin (22 and 10 vs. 11 and 21, *p*=0.012) and UVC (64 and 37 vs. 34 and 67, *p*<0.001)

These p-values are now included in the main text, and the description of the calculation is added to the methods.

3) No genetic requirements for collateral mutations in UVC-treated cells are presented. This seems to miss an opportunity to confirm whether multiple TLS polymerases contribute depending on what type of lesion is causing the damage. For example, is pol eta generating collateral mutations in UVC treated cells and potentially melanomas. The data showing pol kappa does not influence collateral mutations in HR-deficient cells seems to support that differences in the initiating lesion determine with polymerase is causing the collateral mutations.

We primarily used cisplatin as model agent to uncover the genetic dependencies of collateral mutagenesis. However, we fully agree with the referee both that the analysis of the genetic dependence upon UVC would be interesting, and that there are likely pathway differences in bypassing cisplatin and UV lesions.

Whole genome sequencing is expensive though, and an additional range of samples was beyond the budget of this study.

4) Similarly, REV1-/- and REV3L-/- mutants were not evaluated due to lethality with the treatments. Could lower doses of cisplatin be used to evaluate their roles in collateral mutations by this sequencing method?

As explained in our response to question 6 of reviewer 3, we would have had to use such low doses that we could not expect any reasonable numbers of mutations.

5) In Fig.5, the authors assess the enrichment of CC and GG sequences near solitary mutations that do not fit the cisplatin substitution signature in cisplatin treated DT40 cells. They show a small enrichment of these sequences suggesting that some of these solitary mutations may result from TLS polymerase recruitment to a cisplatin lesion, which was bypassed error-free, and subsequently TLS made another error. Based on this, the authors try to calculate the percentage of mutations that are collateral TLS errors. They determine the number of solitary, non-cisplatin signature mutations that have a CC dinucleotide within 10 nt upstream of the mutation or a GG dinucleotide within 10 nt downstream and suggest that all of these mutations (17 in total) are collateral TLS mutations to suggest that 12% of non-primary mutations are due to this mechanism. This percentage, however, is likely a significant over-estimation. By random chance, one would expect to find a CC or GG dinucleotide within 10 nt of half of these mutations, which would place the minimal estimate more in the range of 8 or 9 events. The number of these events observed, then becomes very small and may be heavily influenced by random variations.

The reviewer is absolutely correct that CC and GG dinucleotides are also expected to occur randomly around any given set of mutations. We have included these in our calculations, but did not provide a clear description. In the revised manuscript we provide a detailed explanation:

“We found an increased count of CC and GG motifs around these substitutions, in accordance with the directionality of collateral mutagenesis (Fig. 5B). The median frequency of CC or GG dinucleotides in a 300 bp wide window around all 962 non-primary, solitary mutations was 4.57% per position each, but we found within the first 10 positions 1014 CC dinucleotides to the 5’ direction or GG motifs to the 3’ direction, which is significantly more than the expected 880 events (p=0.0013, Fisher’s exact test), meaning on average 16.75 extra such events per genome in the 8 sequenced samples.”

6) The de novo NMF-derived signatures for cisplatin-induced mutations does not appear to be very stable between the analyses in Fig. 3a and Fig. 5c. This often results from utilizing NMF on datasets with small sample sizes and likely introduces significant error in attributing specific mutations to a specific signature and identifying the cause of the signature. This is particularly problematic for trying to parse mutations that are caused by TLS polymerases making errors across from a cisplatin lesion from collateral TLS mutations that are also induced by damage which recruits the TLS polymerase. This makes me question how specific the Sig_CM signature in fig. 5 is for collateral mutations or whether it also includes cisplatin-induced mutations. An experimental validation that Sig_CM is truly a collateral mutation signature would help. Do the pol kappa-/- or PCNA K164R cells lack Sig_CM specifically or are they required for all signatures? This was not assessed in fig. 5d.

The stability of NMF-derived signature derivation is indeed a perennially relevant question. In the present case, Sig_Cisp_2 from Fig5C, which is derived from a DT40-only dataset and which dominates in cisplatin-treated WT DT40 samples, is very similar to Sig_CispC>A from Fig3A, which is derived from a multi-species dataset but also dominates in cisplatin-treated DT40 (cosine similarity = 0.923). The other signatures are not expected to match, as the associated processes are only included in one or the other sample set. We added the following remark to the manuscript: “Sig_Cisp_2 showed high similarity to Sig_CispC>A from the NMF on mixed cell types presented above (cosine similarity: 0.923), and both signatures dominated the mutation set of cisplatin-treated WT cells, illustrating the stability of NMF-based mutational signature derivation.”

While it is true that some random noise may be still present in these NMF components, due to somewhat low sample sizes, we believe that Sig_CM truly represents collateral mutations for three reasons. First, we point out in the text that this component was correctly present in an approximately 50% ratio in the spectrum of mutations from proper collateral mutation pairs in Fig5D, for any given mutagenic effect (cisplatin, UVC, HR-deficiency). Second, no characteristics peaks of the main C>A type cisplatin spectrum (Sig_CispC>A from Fig3A and Sig_Cisp_2 from Fig5C) seems to be specially enriched in Sig_CM, again arguing against the bleed-through of the cisplatin component into Sig_CM. Finally, we also used the reviewer’s suggestion to validate the signature by testing for its presence in samples not used for its derivation. We used the signatures from figure 5C to deconstruct the mutation sets of BRCA1-/-, BRCA1-/- PCNA-K164R and BRCA1-/- POLK cells treated with cisplatin. We opted for the BRCA1 background to have access to larger mutation sets. In the new figure panel 5E we show that the contribution of Sig_CM is significantly lower in BRCA1-/- PCNA-K164R cells compared to the BRCA1-/- controls, validating the dependence of the collateral process on PCNA ubiquitylation. The inactivation of POLK did not cancel the Sig_CM mutations, probably because POLK appeared to have no role in HR deficiency related collateral mutagenesis (Fig. 4D) and thus only a minor role in overall collateral mutagenesis in cisplatin-treated BRCA1 cells (Fig. 4E).

7) The basis for the author’s claim that up to 20% of mutations are collateral mutations is based on the contribution of Sig_CM in specific genomes and if this signature is not specific for only collateral mutations, then that prevalence is a significant over-estimation. I believe an estimation based on the enrichment of CC and GG sequences near solitary, non-primary mutations would be more accurate. Based on the arguments in point 5, it would then be about 3-4% of total cisplatin-induced mutations.

In our opinion, the use NMF to determine the contribution of collateral mutagenesis to the overall mutation counts can be justified by two ways. First, as it can be seen in Fig5D, the contribution of Sig_CM to the spectra of all mutations in close pairs was always around 50%, with the other half corresponding to the given sample’s particular mutagenic effect. It means that the spectrum of close mutation can be split up to the primary and the collateral mutations, approximately in a 1:1 ratio, which makes sense, as we have seen that usually a close pair consists of a primary and a secondary, collateral event. However, it will also mean that Sig_CM must represent the pure spectrum of collateral mutations, with some possible noise due to the low number of such mutations.

Second, according to our answer to point 5, we believe that our initial estimation using the GG and CC enrichments was correct. As pointed out in the text, the results of the two methods are in good agreement, lending support to the NMF-based approach.

Minor:

1) On page 5, it is stated that cisplatin, UVC, and BRCA2-deficiency induce close mutation pairs involving one insertion/deletion mutation, but it’s not statistically significant. If it is not significant then how can they be confident that event class is induced. This statement should be removed.

We agree that our phrasing was not clear. Although we are not drawing any further conclusions from these data, we deemed our observations worth mentioning in case readers are interested in further analysis.

In the revised manuscript we made it clear that we are not making a comparison to untreated wild type cells, but that we think it noteworthy that cisplatin and UV induced more mixed close pairs than MMS: “A comparison to untreated wild type cells was not informative due to the very low event counts in those genomes, but the proportion of indels with nearby SBS mutations was significantly higher upon cisplatin or UV treatment than in the case of MMS, mirroring the data from base substitution CMRs (see Fig. S1C-D for details).” We added significance values to the supplementary figure.

2) Indicate that the PCNA-K164R cells have mutations in both alleles.

We indicated the correct phenotype in the Methods and Results sections.

3) The trinucleotide contexts on the x-axis of fig. 2a indicate that all mutations are at C bases. The rightmost half need to be corrected to be in T bases.

We thank the reviewer for this remark, we corrected the figure.

4) The y-axis of fig 3d, 4c, S4c, and S5B just state “frequency”. Specify what ratio is being taken. Similarly, Fig 5b and S6 just state CC or GG frequency, but it is unclear what is being divided to generate these numbers.

The ambiguous y-axis labels have been changed to more meaningful labels.

5) Fig. S3 and S7 lack y-axis titles.

Y-axis labels have been added to these figures.

**Reviewer #3:** The authors analyze closely spaced mutation pairs in chicken DT40 cells treated with various mutagens or carrying DNA repair defects. They find that cisplatin, UVC, and homologous recombination defects elevate the frequency of closely spaced mutations. They further detect such mutation pairs in human cell lines and melanoma. The analysis is based on sequencing whole genomes of the cell lines with or without mutagenic treatment, deducing “primary” lesions from the known specificity of the mutagen, and quantifying mutations in the vicinity of the presumed primary lesions. The authors aim to provide evidence, in vertebrate cells, for a phenomenon known in bacteria and lower eukaryotes as “untargeted” or “hitchhiking” mutations. The effects of translesion synthesis (TLS) polymerases and PCNA ubiquitination are also studied, in line with the idea that mutations downstream from the lesion must result from TLS. The analysis is technically sound, and the paper is well written for the most part. This reviewer, however, feels that the study is more appropriate for a specialized journal. The concept of multiple mutations has been extensively studied. While the analysis confirms their occurrence in chicken and human cells, it provides limited information on the mechanisms of these mutations.

Major comments:

1) It is generally known that mutagenic treatments induce closely spaced mutations. While the authors argue that studies in vertebrate cells are limited, the genome-wide analysis presented here is complicated due to the inaccuracy of predicting primary lesion sites (see below). The insight into the mechanisms of multiple mutations from this study is also limited due to a lack of adequate approaches (also see below). Thus, the conclusions hardly go beyond re-stating that closely spaced mutations exist.

In the revised manuscript, we inserted several further calculations, clarifications and citations to prove the reliability of predicting primary lesions (see below). We would argue that a phenomenon cannot be considered generally known until it is properly investigated in higher eukaryotic systems. To strengthen our point, we expanded the discussion highlighting the differences between our findings and those from yeast studies.

2) The lesion position is deduced from the type of mutation and the identity of neighbouring nucleotides. This is a major limitation reducing the power of the analysis. As a start, what is the accuracy of defining “primary” mutations? Can one deduce from previous studies what percentage of mutations at true (known) lesions matches the characteristic "primary" mutation type and context? How is the inaccuracy of the prediction accounted for in the analysis of mutation pairs? Without these considerations, it is hard to determine which conclusions are supported by the data and which are not.

The reviewer’s remark is absolutely correct in the sense that the earlier presence of a given lesion cannot be deduced with 100% certainty from a mutation found later. It is exactly the reason why the underlying mutagenic events cannot be easily found for example in HR mutant samples, because the relatively „featureless” SBS3 signature provides no hints for the possible lesion identities.

However, this problem is less severe in the case of cisplatin- and UVC-induced lesions due to the good match of detected mutations to structural evidence of the lesions, hence we restrained our detailed studies to these two mutagenic effects. Both agents have been widely studied for decades, and the structures of the induced lesions are also well known. For example, in the case of cisplatin, both structural (Takahara et al, Nature, 1995), biochemical (Sherman et al, Chem Rev, 1987) and genomic (Hu et al, PNAS, 2016) data support that the intrastrand crosslink lesions only occur at certain well-defined sequence contexts (i. e., two neighbouring purines, most importantly guanines). In a shuttle plasmid mutagenesis assay it has also been showed (Bubley et al, Mol. Carcinog., 1991) that plasmid positions harbouring mutations after cisplatin treatments largely overlapped with sites of lesion-induced polymerase stalling, both occurring at AG or GG dinucleotides. We have added these citations to the Results section that defines primary mutations.

Concordant with the above, the mutations detected after cisplatin treatments show a specific sparse pattern (compared to e.g. SBS3), with the dominant substitution categories occurring opposite adjacent purine bases. The mutation categories marked as primary events on Fig2A (N[C>A]C, N[C>A]T, N[T>A]C and C[T>AN] mutations) were in cisplatin treated cells present with 219.625 +/- 26.27 events (mean +/- SEM), while in mock treated control samples their number was 12.25 +/- 1.7, meaning that on average, 94.4% of these mutations are caused by cisplatin. Furthermore, in a previous publication (Szikriszt et al, Mutagenesis, 2021) we showed that these specific substitution types are associated with cisplatin treatments in a dose-dependent manner. A similar reasoning can be made for UV-related substitutions: mock samples have 8 +/- 1.63, while UVC-treated samples have 1266.67 +/- 161.18 events in the indicated primary mutation categories (i.e., C[C>T]N, T[C>T]N, C[T>A]N and T[T>A]N), suggesting that 99.4% of all assigned primary mutations are directly caused by UVC. We thank the reviewer for raising this point and encouraging these calculations, which we have added to the manuscript text.

The only doubt that may remain is whether there were indeed mutagen-associated lesions present at mutagen-associated mutation sequence motifs. Using a cisplatin example, the question is whether there were GG intrastrand lesion present at mutated GG sites. In the light of the structural and genetic evidence, this is a straightforward conclusion: this is a major cisplatin lesion, this is a major and treatment-specific cisplatin mutation class, and the two match perfectly. Any alternative explanation (purporting that cisplatin specifically induces mutations at GG via a mechanism that does NOT involve GG lesions) is not supported by any evidence.

To answer the reviewer’s last question, we did not see a need for accounting for the inaccuracy of predicting primary mutations as this is likely very low (1-5%). The results, such as the strong directionality of collateral positions relative to the assigned primaries, appear to vindicate this approach.

3) Interactions of DNA polymerases with DNA involve several nucleotides beyond the lesion site. The majority of “collateral” mutations in this study are found within this range. These events are part of the TLS process and do not inform on the extent of error-prone synthesis beyond the lesion. Deducing the “signature” of these collateral mutations is meaningless, as they are not distinct from the events at the lesion itself. A separate analysis is needed for the really close mutations and those further away from the “lesions” (e.g. > 6 nt). The authors acknowledge that close mutations result from the TLS itself in the Discussion, but this is not sufficient. This understanding needs to be incorporated into the overall design of the analysis.

In the following we would like to convince the reviewer that even though many collateral mutations occur near the lesion where their formation could be influenced by the lesion, the analysis of their spectrum/signature is not meaningless.

First, we would like to resolve a possible misunderstanding: we do not aim to define a „general collateral signature” in Fig5, we only used the mutational signature analysis approach to give us an estimation of similar events among solitary mutations. We do not think that Sig_CM in Fig5C is necessarily a universal manifestation of such processes. However, it is able to show the contribution of collateral events to the overall mutagenic burden, at least in our experimental setting: the lack of Sig_CM-associated mutations in cisplatin treated PCNAK164R cells, and the concordance of the absolute number of Sig_CM-associated mutations with our estimation from the CC/GG overrepresentation around non-primary, solitary mutations both support this notion. We qualified our statement in the manuscript that “Sig_CM must be a good representation of collateral mutations in this experimental system.” and also modified the abstract accordingly.

Further, the signature approach demonstrated that the collateral mutation spectrum is similar upon cisplatin or UVC treatment (Fig. 5D, columns with red dots) *even though the primary lesions are different and many of the mutations are near the lesion*. Whilst we agree that multiple mechanisms are likely to be responsible for collateral mutagenesis, our data suggest that all these mechanisms are largely independent of the nature of the lesion. Thus it appears that the slippage of the polymerases is similar whether the primary lesion is due to cisplatin or UVC. This may not be the case for all lesion types, therefore did not wish to over-analyse this point. As described above, we used unsupervised NMF to see if there was a common collateral mutation pattern in our different sample sets, and the meaningful result was positive.

As to the overall design of the analysis, we wished to detect, map and present all mutations that result from TLS activity, unbiased by any theories on their mechanisms of formation. Such data (e.g. fig. 2E) showed that the majority of the mutations occur near the lesion site, an important finding distinct from yeast-based results. Notably, in the analysis we showed that several different mechanisms of collateral mutagenesis are likely to operate: the mutations with slippage were almost exclusively enriched in close distances, while this was not true for the more distant collateral events (numerical data are shown on fig. 3C). One can imagine that distorted double stranded DNA can lead to the slippage of the new strand. In the revised manuscript, we highlighted this idea more prominently in the Discussion. Thus our analysis was able to separate potential mechanisms, but also showed that close and more distant collateral mutations both depend on TLS (fig. 2E). We could not incorporate the slippage classification into the analysis of HRD-dependent close mutation pairs, because we cannot identify the primary mutations in those pairs.

4) The lack of studies in Pol zeta- and Rev1-deficient cells limits mechanistic insight. The severe sensitivity of these cells to the mutagenic treatments indicates that these polymerases play the primary role in the bypass of the respective lesions. While some effects of Pol kappa and Pol eta on very close mutation pairs are observed, these effects are likely secondary and difficult to interpret in the absence of more comprehensive studies of the bypass mechanism. Perhaps analysis of Pol zeta/Rev1-deficient cells and combinations of polymerase defects at lower doses of cisplatin and UVC could provide more insight.

As explained in our response to question 6 of reviewer 1, in order to investigate the effect of mutagenic agents on REV1 and REV3L deficient cells we would have had to use such low doses that we could not expect any reasonable numbers of mutations.

In an alternative approach, we also attempted to investigate the effect of REV1 and REV3L inactivation on spontaneous mutagenesis by whole genome sequencing. The results, which serve as controls in a concurrent manuscript from the lab, indicate that spontaneous mutagenesis decreases by about 50% in these mutants, from abut 60 to 30 mutations per genome in the 50 day experimental period. As the close mutation ratio was no more than about 5% in any of our sequenced samples in this study, the mutation numbers in these TLS mutants do not allow the assessment of collateral mutagenesis on a genome-wide level.

5) Many statements in the text are not supported by statistical analysis of the data.

We included the necessary additional statistical calculations at multiple sites in the manuscript: Fig2F (association between the variant allele and the adjacent bases for collateral mutations), Fig5B (CC/GG enrichments around solitary mutations), for the new figure panel 5E (validation of HRD signature contribution), FigS4B (relationship between close mutation ratios and cisplatin-associated NMF components), and FigS5B (close mutations in melanoma samples).

Minor comments:

1) The first paragraph on p. 3: reference 16 also studied chromosomal UV-induced DNA lesions.

The text citing reference 16 has been updated with this information.

2) Legend to Figure 1, panel A should be revised to avoid the impression that the model was proposed in this work.

The legend of Figure 1A has been changed.

3) What are “consecutive mutations” in Figure 3C?

By “consecutive mutations” we meant those immediate, but distant mutation pairs that are separated by more than 100 bp. The phrasing has been changed in both the label and legend of Fig3C, and also in the respective part of the main text.

**Have all data underlying the figures and results presented in the manuscript been provided?**

Large-scale datasets should be made available via a public repository as described in the *PLOS Genetics* [data availability policy](http://journals.plos.org/plosgenetics/s/data-availability), and numerical data that underlies graphs or summary statistics should be provided in spreadsheet form as supporting information.

Reviewer #1: Yes

Reviewer #2: **No:** The sequencing data is deposited in a public repository and the called mutations are provided in the supplement. However, the numerical data the underlies graphs is only provided for the NMF calculations. To recapitulate the other graphs, someone would need to recount all the corresponding mutations from the mutation list and preform downstream calculations.

Reviewer #3: Yes

We have now provided a Source Data file for all figures.
