# Supplementary material for: Characterisation of the spectrum and genetic dependence of collateral mutations induced by translesion DNA synthesis
Source: PLoS Genet. 2022 Feb 7;18(2):e1010051. doi: 10.1371/journal.pgen.1010051 (PMC8870599; doi:10.1371/journal.pgen.1010051)
Supplement: Attachment — Submitted filename: Response to reviewers.docx [file pgen.1010051.s012.docx]

**Response to reviewers**

We would again like to thank the reviewers for their questions that in every case helped to improve the quality of our manuscript. Our detailed responses for the remaining issues regarding the correct measure of close mutations and source data files are shown below.

**Reviewer #2**: The authors have now sufficiently addressed all of my previous minor concerns. They declined to assess the genetic dependencies of collateral mutagenesis due to UVC light, stating that this would be interesting, but too expensive to conduct the additional sequencing. While needing to avoid increased expense is understandable, it does miss an opportunity to increase the novelty and impact of the work. They additionally state that REV1-/- and REV3L-/- genotypes could not be evaluated due the low dose of cisplatin required to maintain viability would result in very few mutations, which is a reasonable explanation. The clarification of the calculation for the percent of non-primary mutations attributable to collateral mutagenesis is very helpful and supports ~12% of these mutations being caused by this mechanism. The authors also now provide analysis to validate the use of NMF to identify a collateral mutagenesis signature.

One point, however, was not well addressed: the comparison of close mutation ratios instead of the total number of mutation ratios per genome. The authors argue that the use of ratios is more intuitive because cisplatin treatment produces similar numbers of close mutation pairs, but lesser overall mutations as UVC treatment and therefore the difference in spectrum would not be as apparent. While this is true for comparing overall mutation spectra between damaging agents, the use of close mutation ratios to assess differences between different genotypes treated with the same damaging agent is problematic because both the total mutation load and the number of close mutation pairs could be impacted by the change in genotype. This makes it difficult to know if a reduction in close mutation ratio is due to a loss of close mutation pairs or an increase in primary mutations. These analyses should be done for the authors to state their genotypes are specifically impacting the formation of close mutations.

In response to this request by the reviewer, we have provided an analysis of the total number of close mutations for experiments which compared the effect of the same treatment on different genotypes. For full comparability, we prepared a new supplementary figure which shows, side by side, the total number of mutations, the number of close mutations and the close mutation ratios for cisplatin-treated TLS mutants, and for mock and cisplatin treated BRCA1 TLS double mutants (new Fig. S4). We refer to this figure at all appropriate points in the Results section. The extra analysis does not fundamentally change any conclusions:

- We point out that the PCNA-K164R mutant shows fewer close mutations and lower CMR upon cisplatin treatment.

- In case of cisplatin-treated POLK cells, there is indeed a discrepancy between close mutation numbers (no change) and CMR (reduced). The previous version of the manuscript already showed total numbers of mutation pairs on the distance distribution in Fig. 2E, which shows the loss of longer-distance collateral mutations compared to the distribution in WT samples. We adjusted the description of the data to write that “Despite the lower CMR, the absolute number of close mutations was similar in POLK–/– and wild type cells (Fig. S4), but their intermutation distance profile was also different: almost all pairs in POLK–/– cells were separated by 2 bp (Fig. 2E), and especially the mutations copying the adjacent base were missing (Fig. S3C).”

These additional analyses did not alter our conclusions.

We thank the reviewer for the thoughtful criticism. In response, here we would also like to mention that similarly to the CMR-restricted analysis, the analysis of close mutation pairs only could also be misleading. For example, if the primary and collateral mutations are generated by different polymerases largely independently, switching in between, then mutating the polymerase responsible for the primary mutations will reduce the total number of close pairs, because we can only detect collateral mutations next to primary mutations in the main analysis. At the same time, CMR may not change in relation to the remaining, reduced number of primary mutations. Thus it would be a mistake to conclude from this experiment that the affected polymerase is responsible for collateral mutagenesis. On the other hand, if the polymerase responsible for primary mutations also generates collateral events with a given probability, then we would expect that the close mutation numbers and CMRs will be directly proportional, so both measures are equally reasonable to use.

A similar, but converse effect may be seen with cisplatin-treated wild type and POLH mutant cells. In POLH mutants the overall number of mutations is much higher, the number of close mutations is also much higher, but the CMR is almost the same as in WT. From this, one may conclude that pol eta does not affect collateral mutagenesis: it correctly replicates the primary lesions site and then leaves the extension to other polymerases. One would thus expect a similar number of collateral events in WT and POLH cells, except in WT many of these would be solitary mutations near a correctly bypassed primary lesion. And indeed in Fig. 5D it appears that Sig_CM has a higher contribution to the spectrum of solitary mutations in WT than in POLH cells. Thus CMR correctly shows that POLH has little contribution to collateral mutagenesis, even though the number of close mutations changes upon its inactivation.

In the end we did not want to over-interpret the results, but we provide all the data in both raw and processed forms for readers to perform their own analyses.

**Have all data underlying the figures and results presented in the manuscript been provided?**

Large-scale datasets should be made available via a public repository as described in the *PLOS Genetics* [data availability policy](http://journals.plos.org/plosgenetics/s/data-availability), and numerical data that underlies graphs or summary statistics should be provided in spreadsheet form as supporting information.

Reviewer #1: Yes

Reviewer #2: **No:** It does not appear that the numerical data that underlies graphs are provided in spreadsheet form as supporting information.

Reviewer #3: Yes

In response to the first round of reviews, we prepared and uploaded a source data file as supporting information (source_data.xlsx) which contains all numerical data from all figures and supplementary figures. The link to this file was available on page 45 of the combined pdf file prepared by the submissions website. We have now updated this file with data for the new supplementary figure.
